# Forecasting the Spreading of COVID-19 across Nine Countries from Europe, Asia, and the American Continents Using the ARIMA Models

**DOI:** 10.3390/microorganisms8081158

**Published:** 2020-07-30

**Authors:** Ovidiu-Dumitru Ilie, Roxana-Oana Cojocariu, Alin Ciobica, Sergiu-Ioan Timofte, Ioannis Mavroudis, Bogdan Doroftei

**Affiliations:** 1 Department of Research, Faculty of Biology, “Alexandru Ioan Cuza” University, 700505 Iasi, Romania; roxana_20_2006@yahoo.com; 2 Department of Biology, Faculty of Biology, “Alexandru Ioan Cuza” University, 700505 Iasi, Romania; sergiu.ioan.timofte@gmail.com; 3 Leeds Teaching Hospitals NHS Trust, Great George St., Leeds LS1 3EX, UK; ioannis.mavroudis@nhs.net; 4 Laboratory of Neuropathology and Electron Microscopy, School of Medicine, Aristotle University of Thessaloniki, 54124 Thessaloniki, Greece; 5 Faculty of Medicine, University of Medicine and Pharmacy “Grigore T. Popa”, 700115 Iasi, Romania; bogdan.doroftei@umfiasi.ro

**Keywords:** prevalence, incidence, Europe, Asia, the American continents, COVID-19, SARS-CoV-2, epidemiological

## Abstract

Since mid-November 2019, when the first SARS-CoV-2-infected patient was officially reported, the new coronavirus has affected over 10 million people from which half a million died during this short period. There is an urgent need to monitor, predict, and restrict COVID-19 in a more efficient manner. This is why Auto-Regressive Integrated Moving Average (ARIMA) models have been developed and used to predict the epidemiological trend of COVID-19 in Ukraine, Romania, the Republic of Moldova, Serbia, Bulgaria, Hungary, USA, Brazil, and India, these last three countries being otherwise the most affected presently. To increase accuracy, the daily prevalence data of COVID-19 from 10 March 2020 to 10 July 2020 were collected from the official website of the Romanian Government GOV.RO, World Health Organization (WHO), and European Centre for Disease Prevention and Control (ECDC) websites. Several ARIMA models were formulated with different ARIMA parameters. ARIMA (1, 1, 0), ARIMA (3, 2, 2), ARIMA (3, 2, 2), ARIMA (3, 1, 1), ARIMA (1, 0, 3), ARIMA (1, 2, 0), ARIMA (1, 1, 0), ARIMA (0, 2, 1), and ARIMA (0, 2, 0) models were chosen as the best models, depending on their lowest Mean Absolute Percentage Error (MAPE) values for Ukraine, Romania, the Republic of Moldova, Serbia, Bulgaria, Hungary, USA, Brazil, and India (4.70244, 1.40016, 2.76751, 2.16733, 2.98154, 2.11239, 3.21569, 4.10596, 2.78051). This study demonstrates that ARIMA models are suitable for making predictions during the current crisis and offers an idea of the epidemiological stage of these regions.

## 1. Introduction

The outbreak with the new coronavirus (COVID-19) caused by severe acute respiratory syndrome (SARS-CoV-2) has led to a ‘global pandemic’ due to its unprecedented speed of spreading worldwide. Since patient zero that was reported back in mid-November, over ten million people from two hundred and sixteen territories were identified as SARS-CoV-2-infected patients [1].

Significant discoveries have been made in this context during these last nine months. During this period, the clinical panel has been established [2,3,4,5,6,7,8,9]. However, the early studies have also revealed a low [3,4,5,10], up to medium [6,7,8,9] incidence of gastrointestinal deficiencies. The most common symptom was diarrhea [11,12,13,14], which suggests a potential route of action of COVID-19 at the level of the digestive tract.

Unfortunately, until the 10th of July 2020, more than half a million people have died, predisposition being higher in people suffering from chronic diseases and, especially elderly [15]. However, the number of people confirmed positive varies due to finite capacities in epidemiological surveillance between countries.

It can be said without a shadow of a doubt, that this member of the zoonotic coronavirus family has spread over the entire world until the present day. Given that scientists are in a fight against the clock, the need for a sustainable and reliable strategy for planning health infrastructure to control the spread is crucial. This need is all the more imperative as there is no SARS-CoV-2 treatment/vaccine [15].

Modeling daily cases are pivotal for management and future directions. Estimating COVID-19 possible evolution or regression through mathematical and statistical models is groundbreaking to determine short and long-term case estimates. Such approaches are viable not only to predict the COVID-19 spreading course, but also to allocate the resources necessary to restrict the virus spreading [15].

Distinct approaches have been applied with relatively high accuracy for different prediction purposes. Some examples are represented by statistical methods aiming to predict epidemic cases. These include time series [16], or simulation models [17,18], multivariate linear regression [19], backpropagation neural network [20,21,22], and gray forecasting [23,24].

Any epidemiology evolution is defined and influenced by different factors, more precisely by a tendency of randomness. Retrospectively, the usage of the statistics tools above-mentioned are insufficient for analysis and are difficult to generalize. This is why the Automatic Regressive Integrated Moving Average (ARIMA) model has been successfully applied at a much larger scale in various fields, mainly due to its easy-to-use concept and utility algorithm [25].

Therefore, the present study aims to estimate the prevalence trend in Ukraine, Romania, the Republic of Moldova, Serbia, Bulgaria, and Hungary as Central European countries. Moreover, we will also consider the most affected countries presently, such as USA, Brazil, and India.

## 2. Materials and Methods

### 2.1. Data

The daily prevalence data of COVID-19 was taken from The Ministry of Internal Affairs of Romania (https://www.mai.gov.ro/informare-covid-19-grupul-de-comunicare-strategica), World Health Organization (WHO) (https://covid19.who.int/?gclid=CjwKCAjwi_b3BRAGEiwAemPNUYzgrAMkQXN5Z848tjCmGZLJecod03yWxqW_bN248wjgdezXeYg0RoCeFcQAvD_BwE), and European Centre for Disease Prevention and Control (ECDC) (https://www.ecdc.europa.eu/en). MS Excel was used to build a time-series database. Descriptive statistics of the COVID-19 data for the established intervals (10 March and 10 July) are given in Table 1. In order to create an optimum ARIMA model, at least 30 observations are needed [26].

Data analyzed corresponds to the period between 10 March and 10 July. The data set was used to perform and analyze a case estimation model by applying ARIMA that could help us to predict the SARS-CoV-2 evolution in the future.

Therefore, for this study, a time series containing at least 45 data was used to predict COVID-19 prevalence in six Central and Eastern European countries (Romania, Bulgaria, Serbia, Ukraine, Republic of Moldova, and Hungary) was conducted. Furthermore, the same concept was applied for one country from South America (Brazil) and North America (United States of America), and one from South Asia (India) over the next fourteen days with 95% relative confidence intervals (CI).

**Figure 1:**
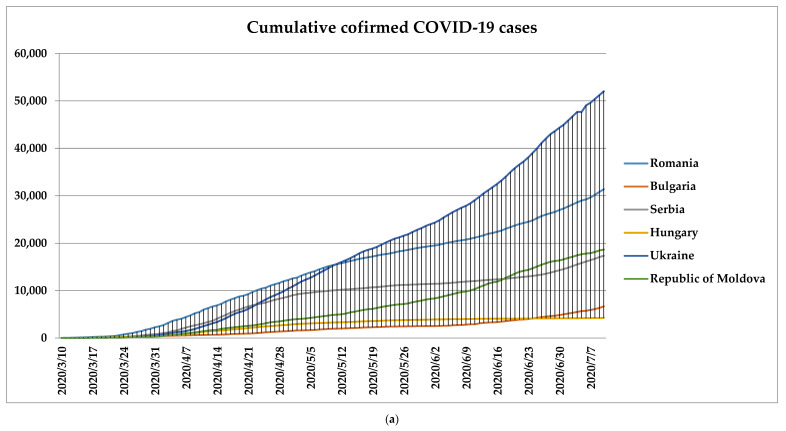

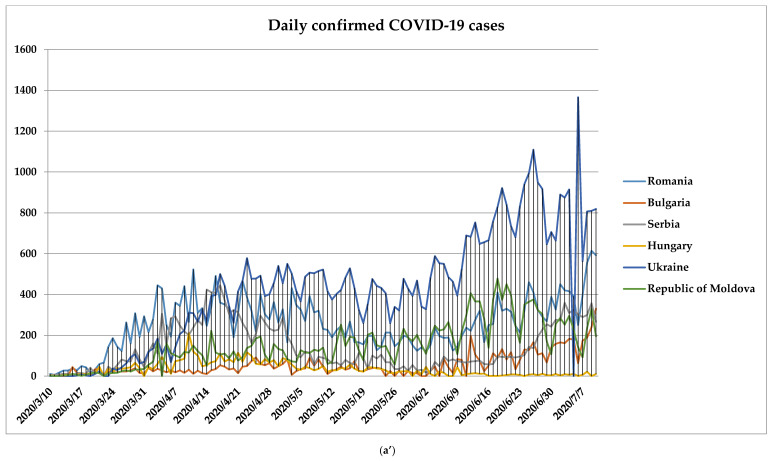

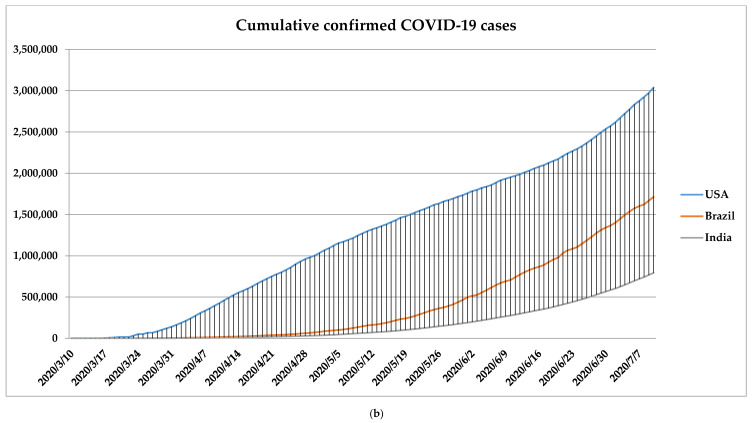

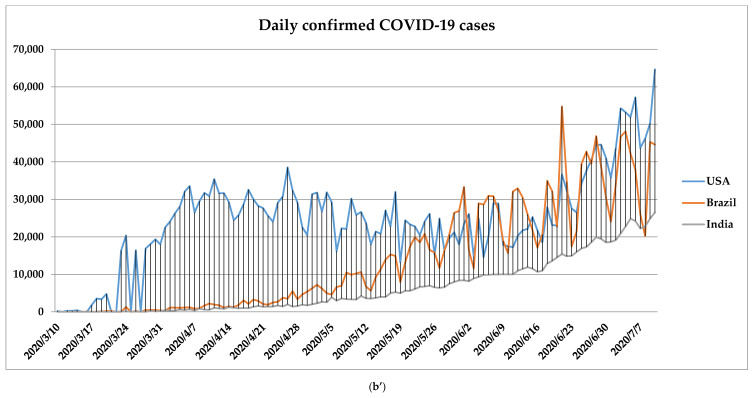
The (**a**,**b**) prevalence and (**a’**,**b’**) incidence of the COVID-19 within the established countries.

As seen from Figure 1, the COVID-19 outbreak hit Ukraine harder than the other five countries between the established period. The first case in Ukraine was reported on 3 March 2020. In contrast with the related regions, the COVID-19 pandemic had started earlier in Romania (26 February) and later in the other four (4 March in Hungary, 6 March in Serbia, 7 March in the Republic of Moldova, and 8 March in Bulgaria). In Ukraine, the total number of confirmed cases of COVID-19 reported during the period is 52,043, the highest number of new cases reported being 1366 registered on 6 July.

The overall prevalence for Romania was 31,381, the second hardest-hit region, followed by the Republic of Moldova with 18,666, Serbia with 17,342, Bulgaria with 6672, and Hungary with 4220 cases. Analogous, the second highest incidence between the remaining five regions was in Romania with 614 new cases in 9 July, followed by the Republic of Moldova with 478 on 18 June, 445 in Serbia on 17 April, 330 in Bulgaria on 10 July, and 210 in Hungary on 10 April.

On the other hand, the first case reported in the USA took place on 20 January, almost one week later compared with Romania. The second hardest-hit region was Brazil, where the first case was reported on 26 February, while in India on 30 January. The overall prevalence for these three countries is as follows: USA with 3,038,325, Brazil with 1,713,160, and India with 793,892 cases. Concerning the incidence, the highest was as expected in USA with 64,630 on 10 July, followed by Brazil with 54,771 on 21 June, and last, India with 26,506 on 10 July.

### 2.2. ARIMA Models

A time series is simply a series of time-dependent data points [27] used for analyses dedicated to revealing reliable and meaningful statistical data for the subsequent prediction of values of a series [28]. Since it was introduced by Box and Jenkins approximately half a century ago, ARIMA began to be used at a much larger scale [26].

In most cases, ARIMA is used since it takes into account all trends and periodic changes, even random disturbances. Thus, ARIMA is suitable for a large spectrum of data, from seasonality to cyclicity. In this context can be modeled a temporal dependency in a flexible manner.

Non-seasonal ARIMA models are defined by three parameters (*p*, *d*, *q*) where *p* is the order of autoregression, *d* is the degree of differencing, and *q* the order of moving average [29]. ARIMA offers the possibility to be modified so that can be conducted different and simple AR, I, or MA models.

AR (p) usually explains the present value Y_t,_ unidirectionally it terms of its previous values Y_t−1_, Y_t−2_, ..., Y_t−p_, and the current residuals ε_t_. MA (q) refers to the current value of the time series Y_t_ in terms of its current and previous residuals ε_t−1_, ε_t−2_,…, ε_t−𝑞_. The general formula of AR (p) and MA (q) can be expressed in Equations (1) and (2).

Y_t_ = Φ_1_Y_t−1_ + Φ_2_Y_t−2_ + … + Φ_p_Y_t−p_ + ε_t_ (1)

Y_t_ = θ_1_ ε_t−1_−θ_2_ ε_t−2_−… θ_p_ ε_t−p_ + ε_t_ (2)

where:

p—past value;

Φ and θ—parameters that indicate the autoregression, and moving average, respectively;

t—time;

Y_t_—observed value at a time t;

ε_t_—value of the random shock dependent by t;

p—past value.

In other words, ARMA (p, q) model expresses the current values, as well as its previous ones and residuals linearly. The corresponding formula is given in the below equation:

Y_t_ = α + Φ_1_Y_t−1_ + Φ_2_Y_t−2_ + … + Φ_p_Y_t−p_ + ε_t_ − θ_1_ ε_t−1_ − θ_2_ ε_t−2_ − …θ_p_ ε_t_ − q (3)

where:

α—constant;

ε_t−1_—value of the previous random shock.

### 2.3. Model Selection

In the present study, three performance criteria entitled Root Mean Square Error (RMSE), Mean Absolute Error (MAE), and Mean Absolute Percentage Error (MAPE) were applied to test the predictive accuracy of the current ARIMA model. Mathematically, the equations for these three criteria are presented above:

(4) $$RMSE=\sqrt{\frac{1}{n}}\overset{n}{\underset{t=1}{\sum }}e_{t}^{2}$$

(5) $$MAE=\frac{1}{n}\overset{n}{\underset{t=1}{\sum }}\left|e_{t}\right|$$

(6) $$MAPE=\frac{100\%}{n}\overset{n}{\underset{i=1}{\sum }}\left|\frac{e_{t}}{y_{t}}\right|$$

where:

y_t_—value observed at a time t;

e_t_—difference between values;

n—number of time points;

For a better fit of the data, RMSE, MAE, and MAPE must have low values. All analyses were performed using STATGRAPHICS Centurion (v.18.1.13) software with a statistically significant level of *p* < 0.005.

## 3. Results and Discussion

### Forecasting the Prevalence of COVID-19 Pandemic Using the ARIMA Model

**Figure 2:**
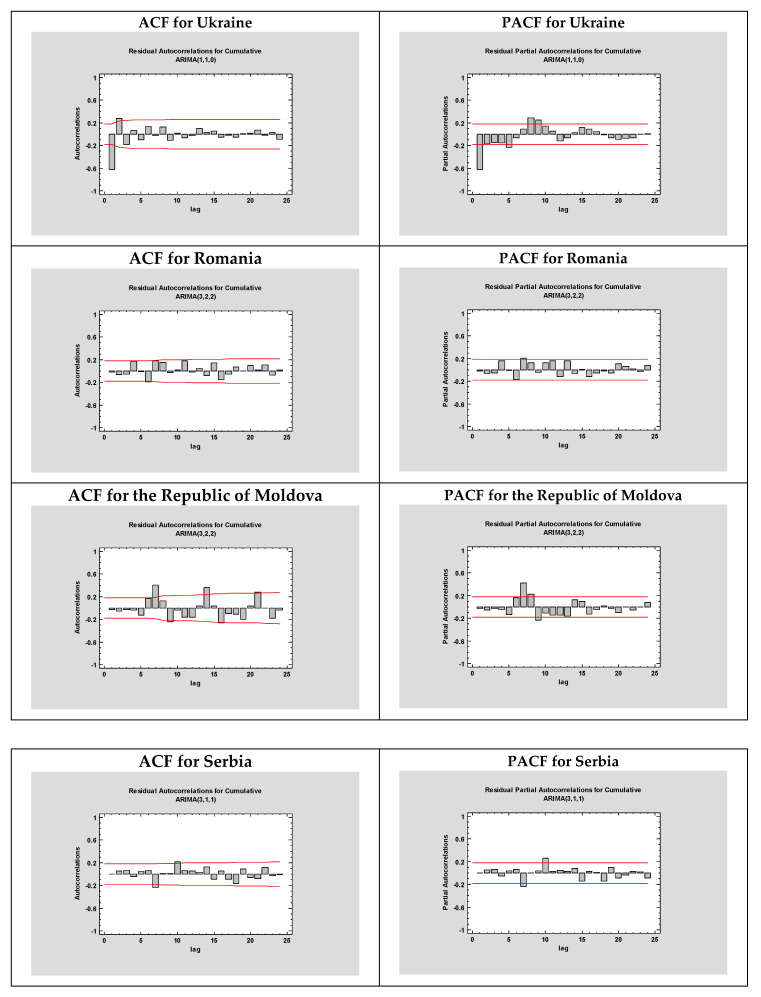

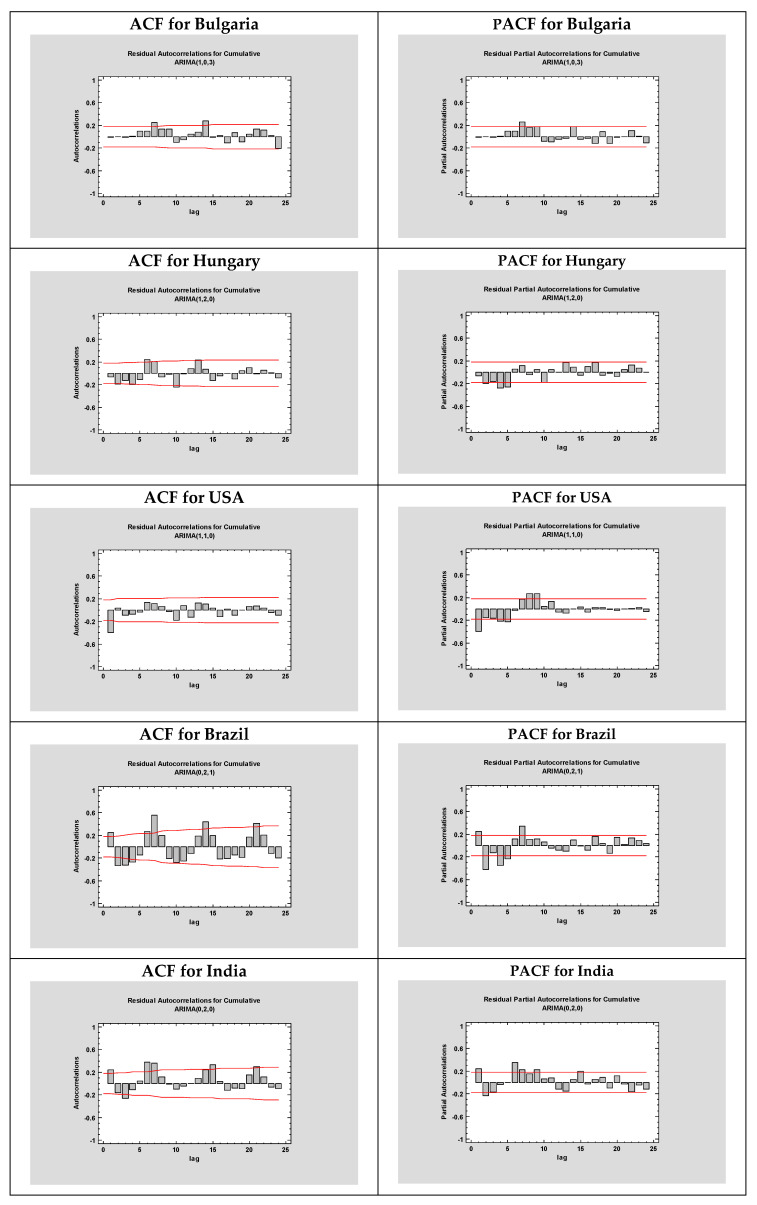
The estimated Autocorrelation Function (ACF) and Partial Autocorrelation Function (PACF) graphs to predict the epidemiological trend of COVID-19 prevalence for Ukraine, Romania, the Republic of Moldova, Serbia, Bulgaria, Hungary, USA, Brazil, and India.

**Figure 3:**
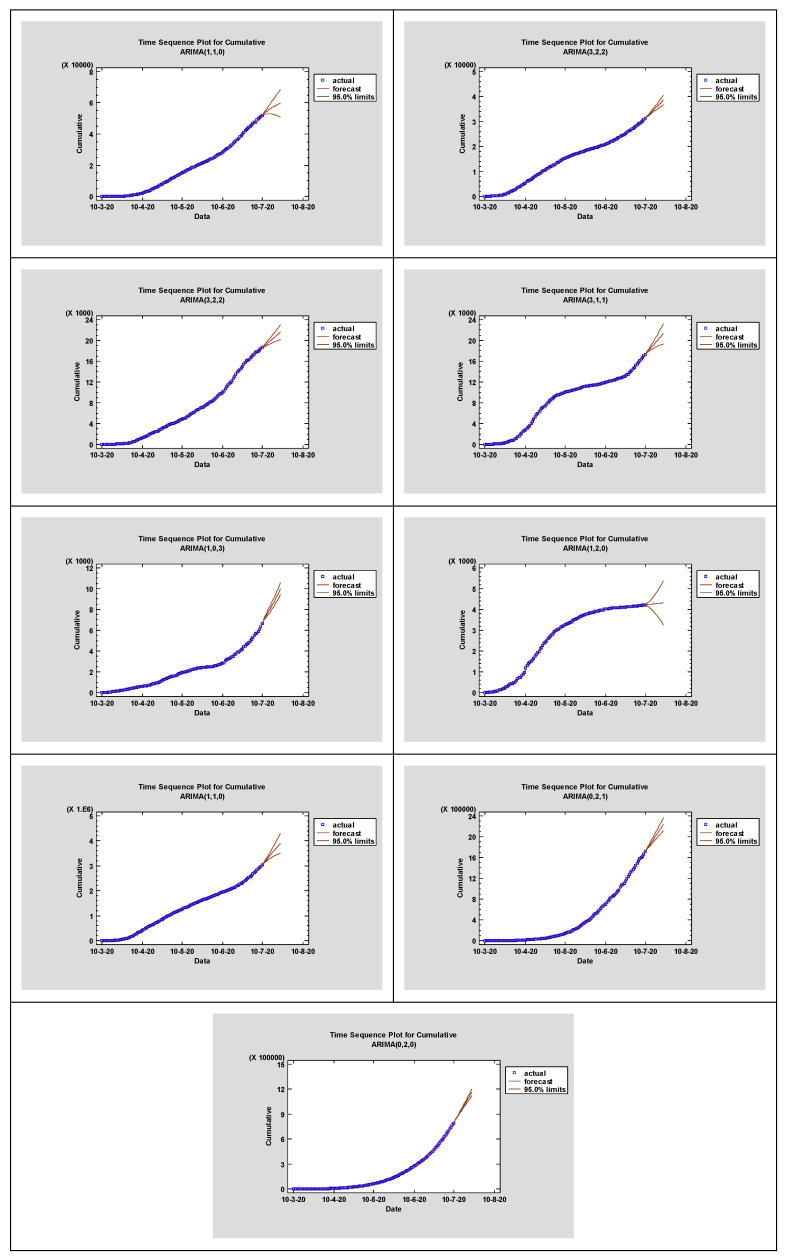
Time-series plots for the best ARIMA models.

The ARIMA modeling is composed of four repetitive steps: assessment of the model, estimation of parameters, diagnostic checking, and prediction. The first step is to control whether the time series’ mean, variance, and autocorrelation constancy over time are stationary and seasonal for a better accuracy [30]. In this context, Time Series plot, Autocorrelation Function (ACF), and Partial Autocorrelation Function (PACF) (Figure 2) graphs were constructed to verify the seasonality and stationarity. On one hand, ACF can determine whether the previous values from the series are related to the following one, while PACF highlights the degree of correlation between a variable and a lag of the said variable [31]. Estimated autocorrelations for the time series of the established countries are shown in Figure 3. Straight lines represent two standard deviations limits, while bars that extend beyond the lines indicate statistically significant autocorrelations.

Additionally, a series of ARIMA models have been also created, and their performances were compared using various statistical tools. All statistical procedures were performed on the transformed COVID-19 data. ARIMA models with the minimum MAPE values were selected as the best model. Among the tested models, the ARIMA (1, 1, 0), ARIMA (3, 2, 2), ARIMA (3, 2, 2), ARIMA (3, 1, 1), ARIMA (1, 0, 3), ARIMA (1, 2, 0), ARIMA (1, 1, 0), ARIMA (0, 2, 1), and ARIMA (0, 2, 0) models were chosen as the best models for Ukraine, Romania, the Republic of Moldova, Serbia, Bulgaria, Hungary, USA, Brazil, and India. The models fitted the COVID-19 data are presented in Figure 2 and Table 2 and Table 3 with a minimum *MAPE_Ukraine_* = 4.70244, *MAPE_Romania_* = 1.40016, *MAPE_Republic of Moldova_* = 2.76751, *MAPE_Serbia_* = 2.16733, *MAPE_Bulgaria_* = 2.98154, *MAPE_Hungary_* = 2.11239, *MAPE_USA_* = 3.21569, *MAPE_Brazil_* = 4.10596, *MAPE_India_* = 2.78051.

Table 3 shows the parameter estimates for the best models. The *p*-values of the associated with the parameters are less than 0.005, so the terms are considerably different from zero at the 95.0% CI. The fitted and predicted values are presented in Figure 3. As seen in Table 4, the next 14-day estimate of confirmed cases may be between 52,816–59,679 in Ukraine, 31,838–38,650 in Romania, and 18,836–21,601 in the Republic of Moldova, 17,639–21,313 in Serbia, 6931–10,000 in Bulgaria, 4225–4319 in Hungary, 3.10259 × 10^6^–3.90611 × 10^6^ in USA, 1.75087 × 10^6^–2.24113 × 10^6^ in Brazil, and 8.20308 × 10^5^–116,489 × 10^6^ in India, respectively.

In the present study, an ARIMA model has been selected, in which the best model forecast for future data is given by a parametric model relating the most recent data value to previous data values and previous noise, or residuals in this context. The output summarizes the statistical significance of the terms in the forecasting model. Terms with *p*-values less than 0.05 are statistically significantly different from zero at the 95.0% confidence level. The *p*-value for the AR(x) or term is less than 0.05, so it is significantly different from 0. The *p*-value for the MA(x) term is less than 0.05, so it is significantly different from 0. When the trend is increasing, in order to obtain a linearity or central trend, the model also chooses q. The estimated standard deviation of the input white noise depends on the best model that was selected during the simulations performed.

According to the current literature, this would be the first study of such a manner. Therefore, the idea of a cluster of nations, and the rate of the spread between them is novel. This adds to the fact that this is the first study to address the situation of the most affected nations globally. In the present study the current situation of the COVID-19 pandemic in Ukraine, Romania, the Republic of Moldova, Serbia, Bulgaria, Hungary, USA, Brazil, and India was presented, and the ongoing trend and extent of the outbreak were estimated by the ARIMA model. According to our best of knowledge, this study is the first of its kind to implement ARIMA models to predict the prevalence of COVID-19 in such a manner.

In the current literature can be found limited data regarding the usage of ARIMA for the prediction of the COVID-19 course. Most reports evaluated the situation from western and southern Asia. Reports regarding the status of Europe are elusive for an unknown reason, and as a consequence, Europe gradually become the second mainland (Table 5). It should be also mentioned that papers that have been subjected to the peer-review process were excluded.

Effective strategies are now all more imperative to control the spreading of COVID-19. Thus, estimating epidemiological trends is crucial for the allocations of medical resources and production activities.

Among the most effective alternatives that proved their efficacity is quarantine. Chintalapudi et al. [34] have discussed the beneficial impact lockdown had within the Italian population in terms of transmissibility. A data-driven model analysis demonstrated a decrement up to 35% of total registered cases, concomitantly with an increase up to 66% of recovered cases after lockdown and self-isolation. The accuracy of these two parameters was 93.75 and 84.4%, respectively.

This tendency of regression proved to be true according to the results obtained by another group of authors. The accuracy of six performance metric models has been tested. Long short-term memory (LSTM) was found to be the most accurate during the study, perspective predictions within the next two weeks being made. Thus, is expected a slight decrease in the number of the total cumulative cases [35].

These observations are strengthened by the results of Papastefanopoulos et al. [40]. Six different time series approaches were also utilized to test the accuracy concerning the COVID-19 outbreak for the top ten most affected countries. Machine learning time series methods were efficiently used to estimate the percentage of the population that will be affected.

By using a stochastic modified SEIR model (susceptible–exposed–infectious–recovered) and due to lack of effective pharmaceutical interventions against SARS-CoV-2, López et al. [41] concluded that social confinement should remain in place for the next two months. Behavior, awareness, and immunity decay is attributed to 99% of the current wave. The gradual incorporation of up to 50% of daily working proportion should be also considered.

It has been recently shown that Black and South Asian people are more prone to infection and subsequently death than the rest. Among the risk factors is age, being male, deprivation, diabetes, asthma, and numerous other medical conditions following the analysis of a cohort consisting of 17,278,392 UK individuals [42].

If all these restrictions are not respected, humanity will face a second wave of infections much more severe than the previous one [37] according to the latest statistics reported by WHO. Most certainly, governments’ internal politics and capability in managing the current situation would be definitory during this temporary crisis [33,36,37,38].

Assuming that 20% of the population of each country in the US will be infected, age-specific mortality pattern shown that counties will be probably heavily affected. These findings suggest the adequate allocation of the medical care resources per capita needed to outside communities to restrain the spread [43].

Chakraborty et al. [32] revealed that to people over the age of 65 should be paid more attention, which is why for them it is recommended intensive care and isolation. In addition, they suggests that the locktime period must be extended, in parallel with the arranging medical centers by increasing the number of beds.

Furthermore, Demongeot et al. [39] have brought a new perspective regarding the important role temperature has on COVID-19 spreading, reflected by the total number of active cases. It seems that high temperature directly reduces contagion rates, but this does not mean seasonal temperature could not support the later reappearance following the usage of time series methods.

## 4. Conclusions

Forecasting the prevalence of a disease is crucial for health departments to create an optimum environment and conditions for patients. As has been presented throughout this manuscript, time series models play an important role in disease prediction. In this study, ARIMA time series models were successfully applied to estimate the overall prevalence of COVID-19 in nine countries, six of them being neighbors, while the other three are the most affected today.

## Figures and Tables

**Table 1:** Descriptive statistics on the prevalence and incidence of coronavirus (COVID-19) in the established countries.

**(a) Prevalence**								
**Continents**	**Country**	**Mean**	**SE Mean**	**St. Dev**	**Minimum**	**Maximum**	**Skewness**	**Kurtosis**
Central and Eastern Europe	Ukraine	17,545.34	1424.02	15793.16	1	52,043	0.5836	−0.8257
	Romania	13,958.65	837.07	9283.60	25	31,381	−0.0447	−1.1718
	Republic of Moldova	6341.02	516.67	5730.20	3	18,666	0.6936	−0.7263
	Serbia	8159.77	468.22	5192.87	5	17,342	−0.3619	−1.1779
	Bulgaria	2063.34	151.29	1677.90	4	6672	0.7943	−0.0721
	Hungary	2618.48	138.75	1538.90	12	4220	−0.5892	−1.2725
South and North America, and South Asia	USA	1,242,336.35	80,297.41	890,541.35	696	3,038,325	0.1309	−1.1182
	Brazil	405,199.86	45,415.32	503,680.30	25	1,713,160	1.1576	0.0707
	India	168,929.42	19,430.70	215,496.91	50	793,802	1.3365	0.7176
**(b) Incidence**								
**Continents**	**Country**	**Mean**	**SE Mean**	**St. Dev**	**Minimum**	**Maximum**	**Skewness**	**Kurtosis**
Central and Eastern Europe	Ukraine	423.10	25.90	287.33	0	1366	0.3931	−0.0052
	Romania	255.00	11.65	129.25	6	614	0.2298	−0.0560
	Republic of Moldova	151.74	10.03	111.27	0	478	0.7573	0.1075
	Serbia	140.98	10.22	113.38	0	445	0.8188	−0.4346
	Bulgaria	54.21	5.08	56.44	0	330	1.9756	4.8870
	Hungary	34.23	3.09	34.36	0	210	1.6979	4.7398
South and North America, and South Asia	USA	24,697.99	1152.30	12,779.70	0	64,630	0.1378	0.8261
	Brazil	13,927.92	1307.41	14,499.97	0	54,771	0.8961	−0.3030
	India	6453.31	644.93	7152.72	0	26,506	1.1402	0.2809

**Table 2:** Comparison of tested Auto-Regressive Integrated Moving Average (ARIMA) models.

Country	Model	RMSE	MAE	MAPE
Ukraine	(1, 1, 0)	182.403	86.857	4.70244
	(0, 2, 0)	184.534	84.6694	4.75145
	(3, 2, 0)	140.184	87.0874	4.86564
	(3, 0, 0)	140.834	83.9104	5.02043
	(2, 2, 0)	141.809	86.6818	5.08194
Romania	(3, 2, 2)	72.2811	54.8283	1.40016
	(1, 2, 3)	77.4246	57.1017	1.45906
	(2, 2, 3)	74.5154	55.2809	1.48125
	(3, 2, 3)	76.4564	56.4977	1.52647
	(2, 2, 2)	78.7986	58.4657	1.53212
Republic of Moldova	(3, 2, 2)	61.1658	43.5817	2.76751
	(3, 2, 1)	60.7849	43.5131	2.77257
	(2, 2, 1)	60.6597	43.5749	2.77809
	(3, 2, 3)	61.6063	43.8593	2.84718
	(2, 2, 3)	61.341	43.8542	2.85937
Serbia	(3, 1, 1)	43.0079	28.8086	2.16733
	(2, 1, 3)	43.0409	29.127	2.17147
	(1, 1, 3)	42.8633	29.1174	2.17271
	(3, 1, 0)	42.8659	28.847	2.17729
	(2, 1, 2)	42.8686	29.1841	2.17814
Bulgaria	(1, 0, 3)	33.4732	23.1431	2.98154
	(2, 0, 2)	33.7635	23.0537	3.04647
	(3, 0, 0)	33.5995	22.8279	3.08918
	(3, 2, 0)	35.4064	23.7486	3.08997
	(2, 2, 2)	78.7986	58.4657	1.53212
Hungary	(1, 2, 0)	23.0452	15.101	2.11239
	(0, 2, 3)	21.7985	13.6316	2.15973
	(3, 2, 0)	22.6729	14.2714	2.16096
	(3, 0, 0)	22.6563	14.488	2.16571
	(2, 2, 3)	21.9873	13.6272	2.16876
USA	(1, 1, 0)	6539.46	4673.82	3.21569
	(0, 2, 0)	6541.2	4710.64	3.2431
	(3, 2, 1)	5818.42	4379.88	3.29508
	(1, 2, 3)	5868.51	4434.36	3.29553
	(2, 2, 3)	5888.31	4430.09	3.29999
Brazil	(0, 2, 1)	6134.91	3838.17	4.10596
	(2, 1, 0)	6493.37	3521.69	4.14127
	(2, 2, 1)	5454.19	3118.73	4.15452
	(1, 2, 0)	6515.51	3598.89	4.16568
	(3, 2, 1)	5457.52	3082.2	4.1698
India	(0, 2, 0)	642.607	416.132	2.78051
	(1, 1, 0)	574.812	376.235	2.7951
	(2, 1, 0)	570.378	373.247	3.06874
	(1, 1, 2)	524.071	358.294	3.19978
	(3, 0, 1)	543.562	358.125	3.29689

**Table 3:** Parameters of ARIMA models.

Country and Best Model	Parameters	Estimate	Standard Error	*t*-Statistic	*p*-Value
Ukraine (1, 1, 0)	AR(1)	0.943844	0.0325404	29.0053	0.000000
Romania (3, 2, 2)	AR(3)	−0.410628	0.103264	−3.97648	0.000122
	MA(2)	−0.758911	0.0916899	−8.27702	0.000000
Republic of Moldova (3, 2, 2)	AR(3)	−0.162489	10.6563	−0.0152482	0.987860
	MA(2)	0.341459	26.5106	0.0128801	0.989746
Serbia (3, 1, 1)	AR(3)	0.241924	1.06252	0.227689	0.820282
	MA(1)	−0.572339	2.98064	−0.192018	0.848058
Bulgaria (1, 0, 3)	AR(1)	1.02769	0.00227845	451.048	0.000000
	MA(3)	−0.267346	0.0937488	−2.85172	0.005128
Hungary	AR(1)	−0.401032	0.0836831	−4.79227	0.000005
USA (1, 1, 0)	AR(1)	0.99441	0.0217047	45.8154	0.000000
Brazil (0, 2, 1)	MA(1)	0.758422	0.0565645	13.4081	0.000000
India (0, 2, 0)	no parameter (s)				

**Table 4:** Prediction of total confirmed cases of COVID-19 for the next fourteen days according to ARIMA models with 95% confidence interval.

**Ukraine ARIMA (1,1,0)**				**Romania ARIMA (3,2,2)**				**Republic of Moldova ARIMA (3,2,2)**			
		**Lower 95%**	**Upper 95%**			**Lower 95%**	**Upper 95%**			**Lower 95%**	**Upper 95%**
**Period**	**Forecast**	**Limit**	**Limit**	**Period**	**Forecast**	**Limit**	**Limit**	**Period**	**Forecast**	**Limit**	**Limit**
11-7-20	52,816.0	52,454.9	53,177.1	11-7-20	31,838.2	31,694.9	31,981.5	11-7-20	18,836.6	18,715.5	18,957.8
12-7-20	53,545.6	52,756.2	54,335.0	12-7-20	32,261.6	32,023.7	32,499.5	12-7-20	19,037.2	18,806.5	19,268.0
13-7-20	54,234.2	52,941.6	55,526.9	13-7-20	32,719.8	32,386.2	33,053.5	13-7-20	19,259.3	18,940.0	19,578.5
14-7-20	54,884.2	53,031.4	56,736.9	14-7-20	33,267.3	32,849.6	33,685.1	14-7-20	19,478.9	19,081.4	19,876.5
15-7-20	55,497.6	53,040.6	57,954.7	15-7-20	33,872.9	33,362.9	34,383.0	15-7-20	19,691.4	19,211.9	20,170.8
16-7-20	56,076.7	52,980.2	59,173.1	16-7-20	34,469.6	33,845.7	35,093.5	16-7-20	19,901.5	19,332.3	20,470.6
17-7-20	56,623.2	52,859.4	60,386.9	17-7-20	35,003.7	34,237.5	35,769.8	17-7-20	20,113.1	19,448.2	20,778.0
18-7-20	57,139.0	52,685.5	61,592.4	18-7-20	35,477.4	34,549.7	36,405.1	18-7-20	20,326.1	19,561.3	21,090.8
19-7-20	57,625.8	52,464.9	62,786.7	19-7-20	35,938.6	34,844.5	37,032.8	19-7-20	20,539.0	19,670.8	21,407.3
20-7-20	58,085.3	52,202.9	63,967.7	20-7-20	36,438.8	35,182.0	37,695.7	20-7-20	20,751.6	19,775.9	21,727.3
21-7-20	58,519.0	51,904.2	65,133.8	21-7-20	36,992.8	35,575.4	38,410.2	21-7-20	20,964.0	19,876.7	22,051.2
22-7-20	58,928.3	51,573.0	66,283.7	22-7-20	37,572.2	35,988.4	39,156.0	22-7-20	21,176.4	19,973.7	22,379.1
23-7-20	59,314.7	51,212.8	67,416.6	23-7-20	38,132.9	36,369.6	39,896.1	23-7-20	21,389.0	20,067.1	22,710.8
24-7-20	59,679.3	50,826.8	68,531.9	24-7-20	38,650.7	36,693.2	40,608.1	24-7-20	21,601.5	20,156.8	23,046.2
**Serbia ARIMA (3,1,1)**				**Bulgaria ARIMA (1,0,3)**				**Hungary ARIMA (1,2,0)**			
		**Lower 95%**	**Upper 95%**			**Lower 95%**	**Upper 95%**			**Lower 95%**	**Upper 95%**
**Period**	**Forecast**	**Limit**	**Limit**	**Period**	**Forecast**	**Limit**	**Limit**	**Period**	**Forecast**	**Limit**	**Limit**
11-7-20	17,639.6	17,554.5	17,724.8	11-7-20	6931.5	6865.22	6997.79	11-7-20	4225.99	4180.36	4271.62
12-7-20	17,927.0	17,765.1	18,088.8	12-7-20	7179.18	7065.11	7293.25	12-7-20	4233.59	4147.54	4319.64
13-7-20	18,214.2	17,956.8	18,471.6	13-7-20	7405.16	7239.7	7570.63	13-7-20	4240.54	4102.74	4378.34
14-7-20	18,501.8	18,135.5	18,868.0	14-7-20	7610.22	7392.89	7827.55	14-7-20	4247.75	4051.78	4443.73
15-7-20	18,786.8	18,300.4	19,273.2	15-7-20	7820.95	7559.81	8082.1	15-7-20	4254.86	3993.94	4515.78
16-7-20	19,072.0	18,454.5	19,689.5	16-7-20	8037.53	7736.95	8338.1	16-7-20	4262.01	3930.38	4593.64
17-7-20	19,355.4	18,597.4	20,113.5	17-7-20	8260.09	7922.85	8597.34	17-7-20	4269.14	3861.35	4676.93
18-7-20	19,638.3	18,730.5	20,546.1	18-7-20	8488.83	8116.76	8860.89	18-7-20	4276.28	3787.29	4765.27
19-7-20	19,919.9	18,854.1	20,985.8	19-7-20	8723.89	8318.28	9129.5	19-7-20	4283.42	3708.46	4858.38
20-7-20	20,200.7	18,968.8	21,432.6	20-7-20	8965.47	8527.2	9403.73	20-7-20	4290.56	3625.12	4955.99
21-7-20	20,480.4	19,075.0	21,885.8	21-7-20	9213.73	8743.44	9684.02	21-7-20	4297.69	3537.49	5057.89
22-7-20	20,759.2	19,173.2	22,345.2	22-7-20	9468.87	8966.96	9970.78	22-7-20	4304.83	3445.76	5163.91
23-7-20	21,036.9	19,263.5	22,810.3	23-7-20	9731.07	9197.81	10,264.3	23-7-20	4311.97	3350.08	5273.86
24-7-20	21,313.7	19,346.4	23,281.0	24-7-20	10,000.5	9436.04	10,565.0	24-7-20	4319.11	3250.6	5387.62
**USA ARIMA (1,1,0)**				**Brazil ARIMA (0,2,1)**				**India ARIMA (0,2,0)**			
		**Lower 95%**	**Upper 95%**			**Lower 95%**	**Upper 95%**			**Lower 95%**	**Upper 95%**
**Period**	**Forecast**	**Limit**	**Limit**	**Period**	**Forecast**	**Limit**	**Limit**	**Period**	**Forecast**	**Limit**	**Limit**
11-7-20	3.10259 × 10^6^	3.08965 × 10^6^	3.11554 × 10^6^	11-7-20	1.75087 × 10^6^	1.73873 × 10^6^	1.76302 × 10^6^	11-7-20	820,308	819,036	821,580
12-7-20	3.1665 × 10^6^	3.13762 × 10^6^	3.19539 × 10^6^	12-7-20	1.78858 × 10^6^	1.76922 × 10^6^	1.80795 × 10^6^	12-7-20	846,814	843,969	849,659
13-7-20	3.23006 × 10^6^	3.18183 × 10^6^	3.27828 × 10^6^	13-7-20	1.8263 × 10^6^	1.79985 × 10^6^	1.85275 × 10^6^	13-7-20	873,320	868,560	878,080
14-7-20	3.29325 × 10^6^	3.2228 × 10^6^	3.3637 × 10^6^	14-7-20	1.86401 × 10^6^	1.83027 × 10^6^	1.89775 × 10^6^	14-7-20	899,826	892,858	906,794
15-7-20	3.3561 × 10^6^	3.26091 × 10^6^	3.45129 × 10^6^	15-7-20	1.90172 × 10^6^	1.86038 × 10^6^	1.94306 × 10^6^	15-7-20	926,332	916,897	935,767
16-7-20	3.41859 × 10^6^	3.2964 × 10^6^	3.54077 × 10^6^	16-7-20	1.93943 × 10^6^	1.89016 × 10^6^	1.98871 × 10^6^	16-7-20	952,838	940,702	964,974
17-7-20	3.48073 × 10^6^	3.3295 × 10^6^	3.63196 × 10^6^	17-7-20	1.97714 × 10^6^	1.91958 × 10^6^	2.03471 × 10^6^	17-7-20	979,344	964,291	994,397
18-7-20	3.54253 × 10^6^	3.36035 × 10^6^	3.7247 × 10^6^	18-7-20	2.01486 × 10^6^	1.94866 × 10^6^	2.08105 × 10^6^	18-7-20	1.00585 × 10^6^	987,679	1.02402 × 10^6^
19-7-20	3.60398 × 10^6^	3.3891 × 10^6^	3.81885 × 10^6^	19-7-20	2.05257 × 10^6^	1.9774 × 10^6^	2.12774 × 10^6^	19-7-20	1.03236 × 10^6^	1.01088 × 10^6^	1.05383 × 10^6^
20-7-20	3.66508 × 10^6^	3.41586 × 10^6^	3.91431 × 10^6^	20-7-20	2.09028 × 10^6^	2.0058 × 10^6^	2.17476 × 10^6^	20-7-20	1.05886 × 10^6^	1.0339 × 10^6^	1.08382 × 10^6^
21-7-20	3.72585 × 10^6^	3.44073 × 10^6^	4.01097 × 10^6^	21-7-20	2.12799 × 10^6^	2.03387 × 10^6^	2.22211 × 10^6^	21-7-20	1.08537 × 10^6^	1.05675 × 10^6^	1.11399 × 10^6^
22-7-20	3.78627 × 10^6^	3.46379 × 10^6^	4.10876 × 10^6^	22-7-20	2.16571 × 10^6^	2.06163 × 10^6^	2.26978 × 10^6^	22-7-20	1.11187 × 10^6^	1.07944 × 10^6^	1.14431 × 10^6^
23-7-20	3.84636 × 10^6^	3.48513 × 10^6^	4.2076 × 10^6^	23-7-20	2.20342 × 10^6^	2.08907 × 10^6^	2.31776 × 10^6^	23-7-20	1.13838 × 10^6^	1.10197 × 10^6^	1.17479 × 10^6^
24-7-20	3.90611 × 10^6^	3.5048 × 10^6^	4.30743 × 10^6^	24-7-20	2.24113 × 10^6^	2.11621 × 10^6^	2.36605 × 10^6^	24-7-20	1.16489 × 10^6^	1.12435 × 10^6^	1.20542 × 10^6^

**Table 5:** Studies conducted to predict COVID-19 spreading in which were used distinct statistical approaches.

Disease	Method(s)	Reference
COVID-19	Hybrid ARIMA-WBF	[32] ^$^
	SutteARIMA	[33] *
	Seasonal ARIMA	[34] *
	ARIMA	[35] *
	NARNN	
	LSTM	
	ARIMA	[36] *
	ARIMA	[37] ^$^
	ARIMA	[38] ^$^
	ARIMA	[39] ^$^
	ARIMA	[40] ^$^
	HWAAS	
	TBAT	
	Facebook’s Prophet	
	DeepAR	
	N-Beats	

^$^ European and non-European countries are included; * strictly European countries included.
